# Transforming Growth Factor Beta Signaling Is Essential for the Autonomous Formation of Cartilage-Like Tissue by Expanded Chondrocytes

**DOI:** 10.1371/journal.pone.0120857

**Published:** 2015-03-16

**Authors:** Adel Tekari, Reto Luginbuehl, Willy Hofstetter, Rainer J. Egli

**Affiliations:** 1 Group for Bone Biology & Orthopaedic Research, Department Clinical Research, University of Bern, Bern, Switzerland; 2 Graduate School for Cellular and Biomedical Sciences, University of Bern, Bern, Switzerland; 3 RMS Foundation, Bettlach, Switzerland; Université de Lyon - Université Jean Monnet, FRANCE

## Abstract

Cartilage is a tissue with limited self-healing potential. Hence, cartilage defects require surgical attention to prevent or postpone the development of osteoarthritis. For cell-based cartilage repair strategies, in particular autologous chondrocyte implantation, articular chondrocytes are isolated from cartilage and expanded *in vitro* to increase the number of cells required for therapy. During expansion, the cells lose the competence to autonomously form a cartilage-like tissue, that is in the absence of exogenously added chondrogenic growth factors, such as TGF-βs. We hypothesized that signaling elicited by autocrine and/or paracrine TGF-β is essential for the formation of cartilage-like tissue and that alterations within the TGF-β signaling pathway during expansion interfere with this process. Primary bovine articular chondrocytes were harvested and expanded in monolayer culture up to passage six and the formation of cartilage tissue was investigated in high density pellet cultures grown for three weeks. Chondrocytes expanded for up to three passages maintained the potential for autonomous cartilage-like tissue formation. After three passages, however, exogenous TGF-β1 was required to induce the formation of cartilage-like tissue. When TGF-β signaling was blocked by inhibiting the TGF-β receptor 1 kinase, the autonomous formation of cartilage-like tissue was abrogated. At the initiation of pellet culture, chondrocytes from passage three and later showed levels of transcripts coding for TGF-β receptors 1 and 2 and TGF-β2 to be three-, five- and five-fold decreased, respectively, as compared to primary chondrocytes. In conclusion, the autonomous formation of cartilage-like tissue by expanded chondrocytes is dependent on signaling induced by autocrine and/or paracrine TGF-β. We propose that a decrease in the expression of the chondrogenic growth factor TGF-β2 and of the TGF-β receptors in expanded chondrocytes accounts for a decrease in the activity of the TGF-β signaling pathway and hence for the loss of the potential for autonomous cartilage-like tissue formation.

## Introduction

Traumatic cartilage defects become often clinically apparent in knee, hip and ankle joints. It has been acknowledged for more than two centuries that cartilage defects do not heal spontaneously [1], which is in contrast to many other tissues in the human body. Instead the defects progress and eventually lead to the development of osteoarthritis [2, 3]. To delay or even to prevent progression to osteoarthritis, therapeutic interventions to treat cartilage defects are required.

Autologous chondrocyte implantation (ACI) and its further developments, such as matrix-associated ACI, represent surgical repair strategies that are currently used in clinics [4, 5]. In a two-step procedure, primary chondrocytes are extracted from articular cartilage of an affected patient, expanded *in vitro* to increase the number of cells, which are subsequently re-implanted at the site of the defect. Those cells are either implanted alone or in combination with a suitable biomaterial, such as collagen type I/III membranes [6] or scaffolds based on polymeric polyglycolic/polylactic acid [7, 8]. The microenvironment of cartilage tissue is essential for the maintenance and stabilization of the phenotype and function of chondrocytes. However, upon isolation of cells from the tissue and expansion in monolayer cultures, this microenvironment is drastically changed from a natural three-dimensional structure to a two-dimensional artificial plastic surface. As the chondrocytes adapt to the new conditions, they begin to proliferate, which leads to a decline in the expression of the cartilage-specific collagen type II (COL2) while the expression of collagen type I (COL1) is induced [9]. Once these cells are implanted, they are expected to fill the defect with cartilage tissue. However, the chondroinstructive potential of the microenvironment within the defect and the cells’ ability to respond and to contribute appropriately to this microenvironment is limited, often leading to the formation of a mechanically incompetent fibrocartilaginous tissue [10, 11].

Three-dimensional high-density pellet cultures have been successfully used as a model to investigate the formation of cartilage-like tissues *in vitro* [12–16]. In this culture system, the chondrocytic phenotype of expanded chondrocytes, as characterized by re-expression of the cartilage matrix proteins COL2 and aggrecan (ACAN) can be restored partially. Recapitulating the two steps of ACI *in vitro* revealed that only cells which have been expanded for a short period of time in monolayer culture retained the potential to form cartilage-like tissue in pellet cultures autonomously, that is in the absence of exogenously added chondrogenic growth factors [17–20]. This potential is progressively lost during cell expansion and is correlated with the number of population doublings (PD) the cells have gone through [18, 21, 22]. In ACI, 200,000–300,000 primary chondrocytes are expanded in monolayer culture to approximately 12 × 10^6^ cells, corresponding to roughly six PD [23]. This is around or beyond the threshold of PD, which still would allow for formation of cartilage tissue in the absence of additional growth factors [24]. This may explain the incompetence of the cells to generate a stable long-lasting cartilaginous repair tissue within cartilage defects.

In order to understand the loss of competence of articular chondrocytes to form cartilage tissue autonomously, many studies investigated the phenotypic changes occurring during monolayer expansion. These changes encompass factors which are involved in the interaction with and contribution to the microenvironment, such as integrins and cadherins [25–27], growth factors and growth factor receptors [25, 28, 29], matrix proteins [22, 25, 26, 29, 30], and ion channels [31]. There is, however, only limited information available how those phenotypic changes interfere with the competence of expanded cells to form cartilage tissue.

After expansion in monolayer culture, when cells have lost the potential for autonomous cartilage-like tissue formation, exogenous chondrogenic factors still can induce cartilage-like tissue formation [32]. Transforming growth factor-β (TGF-β) is one of the many signaling factors that are involved in chondrogenesis [33–36], the maintenance of the chondrocyte phenotype, and in the homeostasis of articular cartilage [37–41]. Chondrocytes both respond to and constitutively express the TGF-β isoforms 1, 2, and 3 [42]. Ligands of the TGF-β family initiate specific signaling cascades by binding to type 1 and type 2 TGF-β receptors (TβR1, TβR2). Within this complex, TβR2 phosphorylates TβR1, which propagates the signal by phosphorylating the transcription factors SMAD2 (Mothers against decapentaplegic homolog 2) and SMAD3 [43, 44].

The loss of the potential for the formation of cartilage-like tissue by expanded chondrocytes can be recovered by exogenous TGF-β [24]. Therefore, we hypothesized that TGF-β signaling elicited by autocrine and/or paracrine TGF-β is essential for autonomous cartilage-like tissue formation and that changes within this pathway during expansion in monolayer culture leads to the dependence on exogenous TGF-β. We showed that the autonomous formation of cartilage tissue in pellet cultures is abrogated by blocking the TGF-β signaling pathway through inhibition of the TGF-β receptor 1 kinase. Moreover, the expression of TGF- β2 and the TGF- β receptors diminished during expansion of the chondrocytes, which was paralleled by the loss of the potential to form cartilage-like tissue autonomously. This suggests that indeed impaired TGF-β signaling is a first step contributing to the loss of the potential of expanded chondrocytes to autonomously form cartilage-like tissue.

## Materials and Methods

### Expansion of articular chondrocytes

The bovine shoulder joints of 2-year-old animals were obtained from a local abattoir (Metzgerei Holzer, Hindelbank, Switzerland) 24 hours after slaughter. Articular chondrocytes were isolated from the cartilage by sequential enzymatic digestion with 2.5 mg/ml pronase (Sigma-Aldrich, Buchs, Switzerland) for 1 h, followed by treatment with 450 μg/ml collagenase P (Roche Diagnostics, Rotkreuz, Switzerland) for 4 h at 37°C [45]. The chondrocytes were seeded at a density of 10^4^ cells/cm^2^ in tissue culture flasks in proliferation medium (Dulbecco’s modified Eagle’s medium [DMEM]/Ham’s F12- nutrient mixture [Gibco, Life Technologies, Zug, Switzerland] containing 10% fetal bovine serum [FBS; Sigma-Aldrich, Buchs, Switzerland], and penicillin/streptomycin [P/S; 100 units/ml and 100 μg/ml, respectively; Gibco, Life Technologies, Zug, Switzerland]).

Near confluence, the cells were harvested using Trypsin/EDTA (Gibco, Life Technologies, Zug, Switzerland) and were re-plated at a density of 5 ×10^3^ cells/cm^2^ until passage 6 (P6). The number of passages refers to the number of time the cells were trypsinized. Twenty-four hours after isolation (at which time point cells were referred to as primary chondrocytes) and after each passage, the cells were cryopreserved and stored in liquid nitrogen for further experiments.

### Pellet cultures

The formation of cartilage-like tissue *in vitro* was investigated in high-density pellet cultures [46]. Briefly, 5 x 10^5^ cells in 0.5 ml of DMEM containing P/S, ITS^+3^, 0.1 mM ascorbic acid-2-phosphate, 0.4 mM l-proline, 100 nM dexamethasone (Sigma-Aldrich, Buchs, Switzerland) with or without 10 ng/ml TGF-β1 (Acris Antibodies, Herford, Germany) were centrifuged at 250 *g* for 5 min in 15-ml polypropylene tubes. The cell pellets were cultured for 3 weeks and the medium was changed twice a week.

### Analysis of gene expression

Pellet cultures were pretreated with 450 μg/ml collagenase P for 3 h at 37°C to release the chondrocytes from the matrix before RNA extraction. Total RNA was extracted using the RNeasy Mini kit (Qiagen, Basel, Switzerland) according to the manufacturer’s instructions. The RNA was reverse transcribed using MLV reverse transcriptase (Promega, Dübendorf, Switzerland). PCR was performed on an ABI 7500 sequence detection system (Life Technologies, Zug, Switzerland). The following Assays-on-Demand were used: alpha 1 chain of COL2 (COL2A1, Bt03251861_m1), ACAN (Bt03212186_m1), COL10A1 (Bt03215581_m1), COL1A1 (Bt03225322_m1), TGF-β1 (Bt04259484_m1), TGF-β2 (Bt03276346_m1), TGF-β3 (Bt03272217_m1), TβR1 (Bt03215459_m1), and TβR2 (Bt04281254_m1). The levels of transcripts were normalized to the expression of β2-microglobulin (B2M, Bt03251628_m1).

### Histology

Pellet cultures were fixed in 4% paraformaldehyde for 4 h at room temperature, dehydrated, and embedded in paraffin. Five-micrometer-thick sections were deparaffinized in xylol and rehydrated in graded ethanol before staining.

#### Safranin O staining

Sections were stained for 10 min in 0.2% Safranin-O and counterstained for 2 min with 0.04% Fast Green.

#### Immunohistochemistry

Antigen retrieval was performed by treating the sections with 0.1% trypsin (Becton Dickinson, Basel, Switzerland) for 20 min at 37°C. Endogenous peroxidase was blocked with Peroxidase-Blocking Solution (Dako, Baar, Switzerland). The sections were incubated with either anti-human type I collagen monoclonal antibody (0.1 μg/ml, clone I-8H5; Abnova, Taipei City, Taiwan) or anti-chicken type II collagen monoclonal antibody (0.1 μg/ml, clone A2–10; Chondrex, Redmond, WA, USA) overnight at 4°C. After washing, the sections were incubated with a secondary antibody directed against mouse/ rabbit antibodies, conjugated to a peroxidase-labeled polymer (Envision Dual Link System-HRP; Dako, Baar, Switzerland) for 30 min at room temperature. Bound antibody was visualized using the peroxidase-specific substrate 3,3′-diaminobenzidine (DAKO, Baar, Switzerland), and the sections were counterstained with Meyer’s hematoxylin.

### Inhibition of the TGF-β signaling pathway

TGF-β receptor 1 kinase was selectively blocked using the kinase inhibitor SB-505124 (Sigma-Aldrich, Buchs, Switzerland). The compound was reconstituted in DMSO to obtain stock solutions of 0, 0.3, 0.75, 1.5, or 3 mM, and added to the monolayer and pellet cultures at concentrations of 0, 100, 250, 500, or 1000 nM, respectively. At each medium change, the culture medium was supplemented with the inhibitor.

### SMAD reporter luciferase assay

The activation of SMAD2/3 was assessed using the Cignal SMAD reporter assay (Qiagen, Basel, Switzerland) according to the manufacturer’s instructions. Briefly, 10^4^ cells were seeded in 96-well plates in the proliferation medium without antibiotics a day before transfection. Cells were transfected with the reporter constructs, using Lipofectamine2000 (Invitrogen, Zug, Switzerland). After 24 h, the medium was replaced with medium containing 0.5% FBS. To inhibit the TGF-β signaling pathway, the cells were pretreated with the inhibitor SB-505124 for 30 min before the addition of TGF-β1 (10 ng/ml). After 20 h, the cells were harvested and assayed for luciferase activity by using a dual luciferase assay, according to the manufacturer’s instructions (Promega, Duebendorf, Switzerland).

### XTT assay

Chondrocytes were plated in 96-well tissue culture plates at a density of 2 × 10^3^ cells/cm^2^ and were grown in the proliferation medium. On day 3, the XTT assay (Cell Proliferation Kit II; Roche Diagnostics, Rotkreuz, Switzerland) was applied as a measure for the number of viable cells, following the recommendations of the manufacturer.

### Statistical analysis

All quantitative data were obtained from up to four animals and are presented with the 95% confidence interval. Statistical differences were evaluated by one-way ANOVA with Bonferroni’s post-hoc test or Student’s t-test using GraphPad Prism version 6 for Windows. A *p*-value of < 0.05 was considered significant.

## Results

### Expansion of chondrocytes and cartilage-like tissue formation in three-dimensional pellet cultures

**Fig 1:**
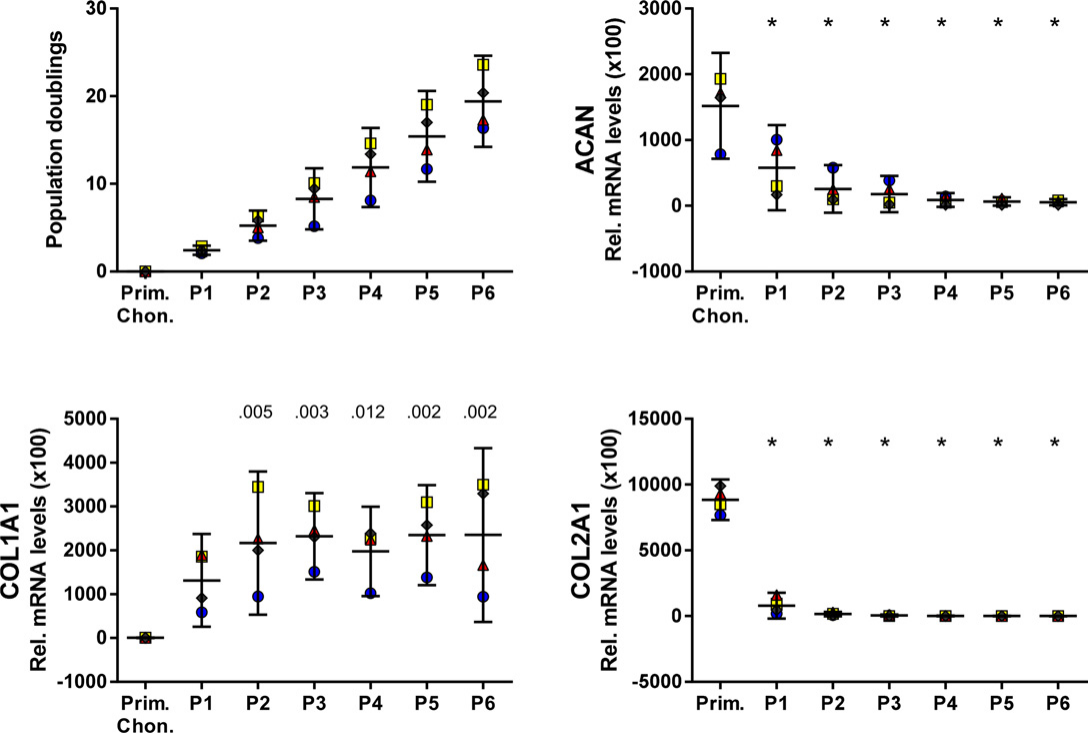
Characterization of expanded bovine articular chondrocytes. Chondrocytes continuously proliferated beyond 20 population doublings. Levels of transcripts encoding cartilage specific proteins COL2A1 and ACAN decreased with increasing passage number, while COL1A1 increased until P2 and remained constant thereafter. Levels of transcripts were normalized to B2M and are presented as mean with the 95% confidence interval derived from four animals (illustrated with different symbols and colors). Levels of transcripts were compared to the levels in primary chondrocytes (Prim. Chon.) (ANOVA), indicated by exact *P* values or * *P* < .001.

Bovine articular chondrocytes were expanded in monolayer culture until passage 6 (P6), corresponding to 19.4 (14.2–24.6) PD (mean (95% confidence interval)) (Fig. 1). Compared with primary chondrocytes, the levels of transcripts coding for COL2A1 and ACAN were more than 1000- and 25-fold decreased, respectively, in P6, while that of COL1A1 showed a 200-fold increase in P1, with a further increase to 370-fold until P6.

**Fig 2:**
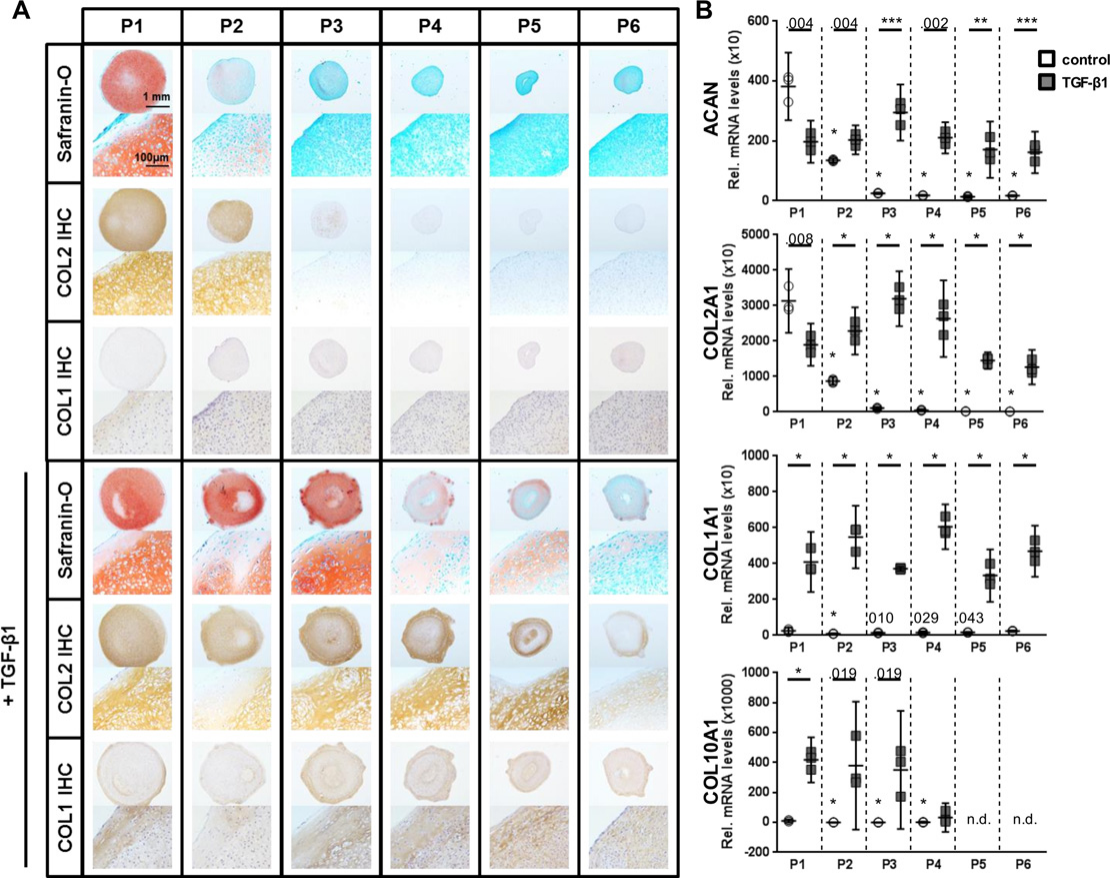
Pellet cultures of expanded chondrocytes. Expanded chondrocytes from P1 to P6 were subjected to pellet cultures without or with TGF-β1. (A) Pellet cultures were analyzed histologically for the presence of glycosaminoglycans (Safranin-O) and COL2 and COL1 proteins (immunohistochemistry [IHC]). Cells from P1, and to a lesser extent from P2, formed cartilage-like tissue autonomously. Addition of TGF-β1 (10 ng/ml) induced formation of cartilage-like tissue in cells from P1 to P6, although a decrease in the staining intensities for glycosaminoglycans and COL2 was observed in cells at P4 and later time points. Cultures treated with TGF-β1 stained more intense for COL1 compared to cultures without TGF-β1. (B) The decreased expression of transcripts coding for cartilage-specific matrix proteins (ACAN and COL2) in pellet cultures from late passages in the absence of TGF-β1 was compensated for by the addition of TGF-β1. TGF-β1 treatment of pellet cultures resulted in an up-regulation of COL10A1 in P1–P4 and increased COL1A1 expression in all passages. Data are shown as mean with the 95% confidence interval from one experiment with three separately analyzed cultures per group. The experiment was repeated with P1–P4 cells and P1/P3 cells from two other animals, respectively, yielding similar results. Levels of transcripts of cultures without TGF-β1 were compared to the levels in P1 without exogenous TGF-β1 (ANOVA). TGF-β1 treated cultures from each passage were compared to the corresponding culture from the same passage without exogenous TGF-β1 (Student’s t-test). Exact *P* values are indicated by numbers or * *P* < .001. n.d.: not detectable.

After each passage, the potential of the cells to form cartilage-like tissue was investigated in high-density pellet cultures with and without exogenous TGF-β1 (Fig. 2A). Only chondrocytes from P1 and P2, which went through less than 5.2 (3.5–7.0) PD, autonomously formed cartilage-like tissue in the absence of exogenous TGF-β1. Reduced intensity of histological staining for glycosaminoglycans (Safranin-O) and COL2 (Fig. 2A, top panel) and decreased levels of transcripts coding for COL2A1 and ACAN (Fig. 2B), however, were observed in P2 as compared to P1 pellet cultures. In pellet cultures from P3 (8.3 (4.8–11.8) PD), the formation of cartilage-like tissue was greatly reduced. In these cultures, the levels of transcripts coding for COL2A1 and ACAN were 33- and 16-fold reduced, respectively, as compared to P1 pellet cultures. Addition of TGF-β1 (10 ng/ml) to the pellet cultures stimulated the formation of cartilage-like tissue by cells from all passages (Fig. 2A, lower panel), although a less intense staining for glycosaminoglycans and COL2 was observed in the pellets from P4 to P6. Compared to P1 pellets, the level of transcripts coding for COL2 and ACAN varied within a factor of 2 in TGF-β1 treated pellet cultures from P2 to P6 (Fig. 2B). Regardless of the culture conditions, in pellet cultures with a matrix containing glycosaminoglycans and COL2 (P1 to P6 in the presence and P1/P2 in the absence of TGF-β1) the levels of transcripts coding for COL2A1 and ACAN varied within a factor of 2 only. In pellet cultures treated with TGF-β1, the expression of COL10A1, a marker for hypertrophic chondrocytes, increased by more than 40- (P1) and 1,000-fold (P2/P3) as compared to the respective cultures without TGF-β1. COL1 was found to be expressed in all pellet cultures, however, in cultures supplemented with TGF-β1 the staining for COL1 protein was more intense and the level of transcripts was 10–60-fold higher as compared to the respective cultures without TGF-β1.

### Blocking of the TGF-β signaling pathway: Effect on the formation of cartilage tissue

Since the loss of the potential for autonomous cartilage formation could be compensated for by exogenous TGF-β1, we pursued the question whether an alteration within the TGF-β signaling pathway during expansion of chondrocytes can be made accountable for these observations. In a first step, the TGF-β signaling pathway was blocked in pellet cultures of P1 and P3 chondrocytes by inhibiting the kinase activity of TβR1 using the inhibitor SB-505124. P1 cells were shown to autonomously form cartilage-like tissue as described above, whereas P3 cells, which required supplementation with TGF-β1 (10 ng/ml), served as a control for the effect and specificity of the inhibitor.

**Fig 3:**
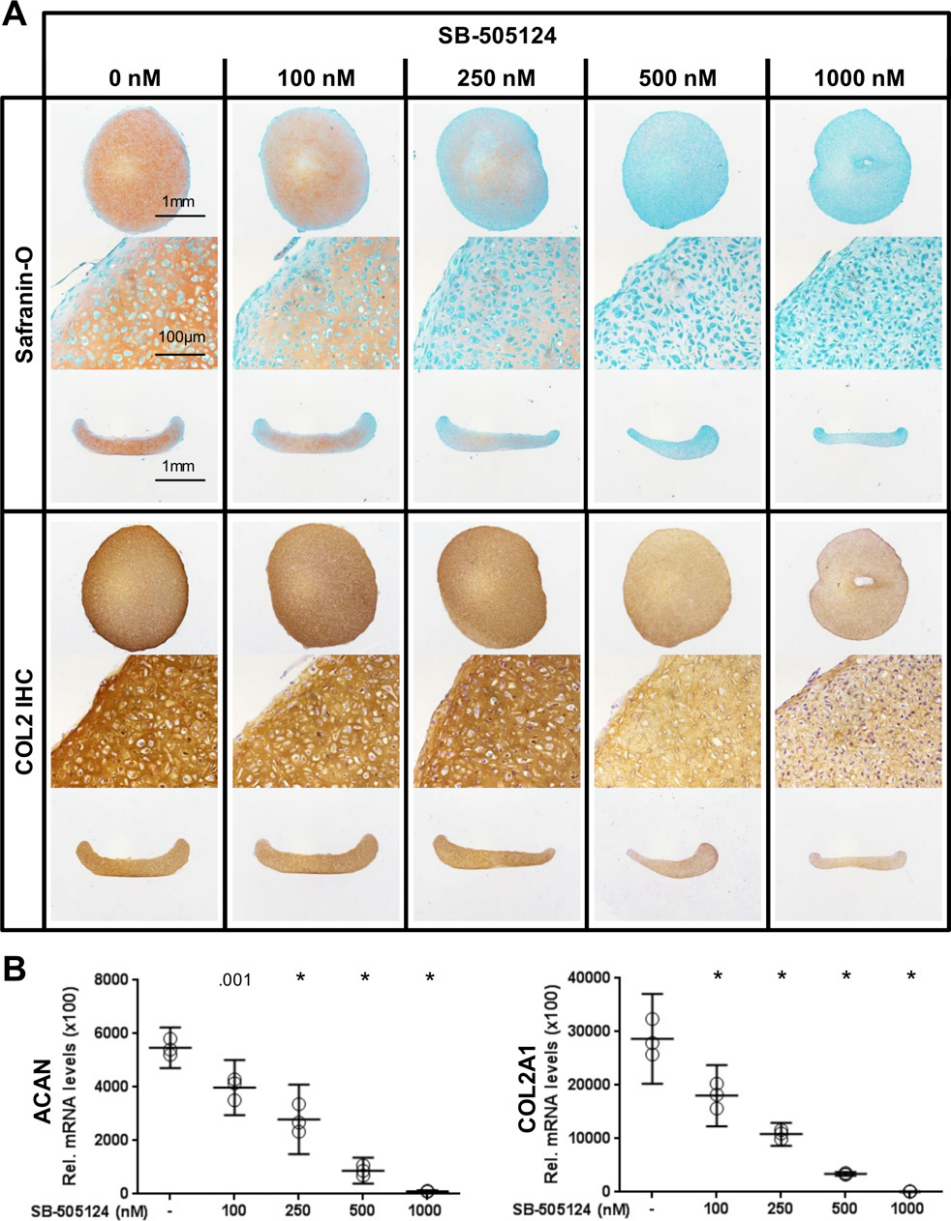
Blocking the TGF-β signaling pathway using the TβR1 kinase inhibitor SB-505124 in pellet cultures of P1 cells. (A) The addition of SB-505124 to the pellet cultures of P1 cells abrogated autonomous cartilage-like tissue formation. Histological sections are shown from perpendicular axes (first and third row of Safranin-O and COL2 immunohistochemistry [IHC]). (B) The levels of transcripts coding for ACAN and COL2 decreased with increasing concentration of the inhibitor SB-505124. Data are shown as mean with the 95% confidence interval from one experiment with three separately analyzed cultures per group. The experiment was repeated once with cells from a different animal yielding similar results. Levels of transcripts of treated cultures were compared to control (0 nM) (ANOVA), indicated by exact *P* values or * *P* < .001.

Inhibition of TGF-β signaling by SB-505124 in pellet cultures from P1 without supplementation with TGF-β1 resulted in a dose-dependent reduction in the deposition of glycosaminoglycans and COL2 in the extracellular matrix (Fig. 3A). The sizes of the pellets formed in the cultures were independent of the concentration of the inhibitor. Furthermore, gene expression analysis revealed a reduction in the levels of transcripts coding for ACAN and COL2A1 in pellet cultures treated with increasing concentrations of the inhibitor (Fig. 3B).

**Fig 4:**
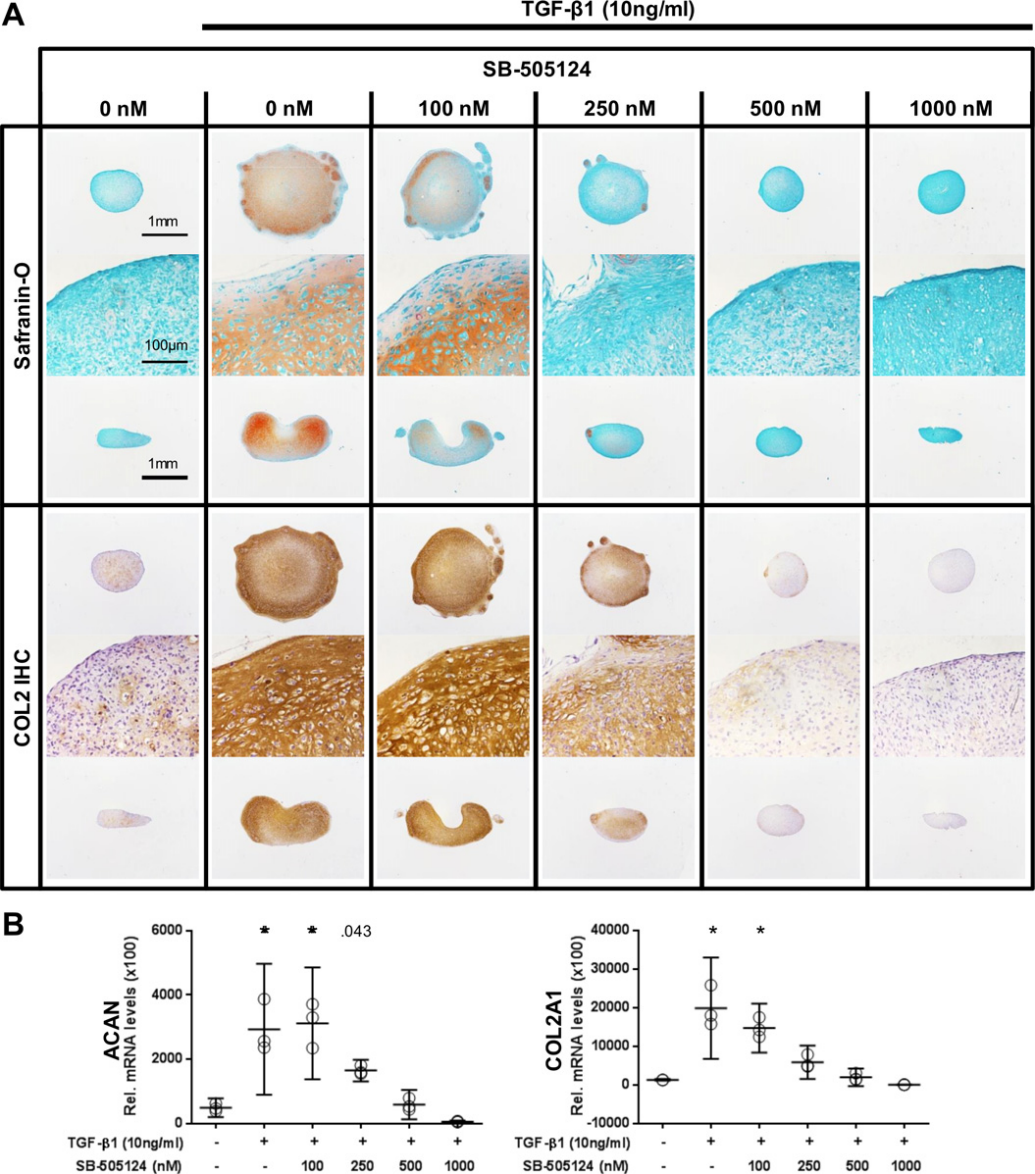
Blocking the TGF-β signaling pathway using the TβR1 kinase inhibitor SB-505124 in pellet cultures of P3 cells. (A) The addition of SB-505124 to pellet cultures of P3 cells abrogated the TGF-β1-induced formation of a cartilage-like tissue. Histological sections are shown from perpendicular axes (first and third row of Safranin-O and COL2 immunohistochemistry [IHC]). (B) The increase in the levels of transcripts coding for ACAN and COL2 induced by exogenous TGF-β1 was abrogated dose dependently by the inhibitor SB-505124. Data are shown as mean with the 95% confidence interval from one experiment with three separately analyzed cultures per group. Levels of transcripts of treated cultures were compared to control without exogenous TGF-β1 (ANOVA), indicated by exact *P* values or * *P* < .001.

While exogenous TGF-β1 allowed for cartilage-like tissue formation in pellet cultures formed with P3 cells, SB-505124 abrogated that effect in a dose-dependent manner, as evidenced by reduced staining intensities for glycosaminoglycans and COL2 (Fig. 4A) and decreased levels of transcripts coding for ACAN and COL2A1 (Fig. 4B).

### Blocking of the TGF-β signaling pathway: Effect on proliferation and TGF-β signal transduction

**Fig 5:**
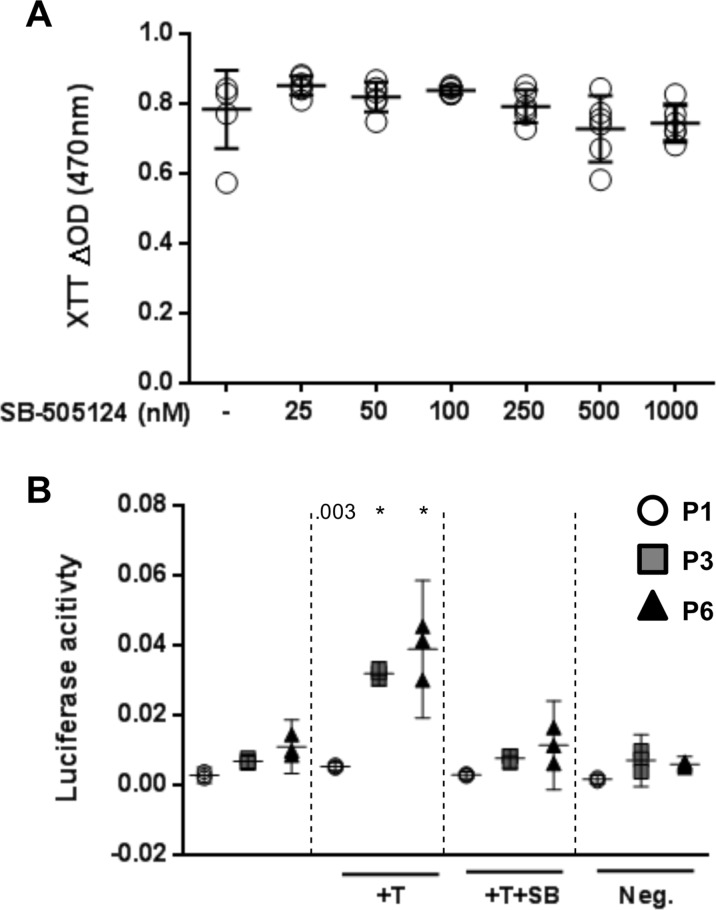
The effect of the TβR1 kinase inhibitor SB-505124 (500nM) on cell viability and activation of SMAD2/3. (A) The number of viable P1 chondrocytes cultured for 3 days in monolayer in the presence of the TβR1 kinase inhibitor was not affected (XTT assay). Data are shown as mean with the 95% confidence interval from one experiment performed in sextuples. (B) Chondrocytes from different passages (P1, P3, and P6) were reactive to exogenous TGF-β1 (T; 10 ng/ml) as evidenced by a SMAD reporter assay. TGF-β1 mediated SMAD-phosphorylation was abolished following the addition of the inhibitor SB-505124 (+T+SB; 500 nM). Neg: negative control (non-inducible reporter construct). Data are shown as mean with the 95% confidence interval from cells of three different animals. Cultures were compared to the corresponding untreated cultures from the same passage (ANOVA), indicated by exact *P* values or * *P* < .001.

To exclude that either a cytotoxic effect and/or inhibition of proliferation by SB-505124 may contribute to the inhibition of autonomous cartilage-like tissue formation, the XTT assay was applied. Up to a concentration of 1000 nM no significant effect of SB-505124 on the number of viable P1 cells was observed, suggesting that the inhibitor is not cytotoxic and doesn’t interfere with cell proliferation (Fig. 5A). P1, P3, and P6 monolayer culture cells were transfected with a SMAD reporter construct to test the potential of exogenous TGF-β1 to elicit intracellular signaling and the ability of SB-505124 to abolish this effect. We found that the SMAD reporter was activated by exogenous TGF-β1 in all three cell populations with a 1.8-, 4.7-, and 3.5-fold induction of luciferase activity in P1, P3, and P6, respectively. The TGF-β1 dependent increase in luciferase activity was abolished by the addition of SB-505124 at a concentration of 500 nM (Fig. 5B).

### Expression of TGF-β isoforms and TGF-β receptors in expanded chondrocytes

We showed that active TGF-β signaling is important for the potential of cartilage-derived cells to autonomously develop a cartilage-like tissue in pellet cultures and that this loss can be partially reversed by exogenous TGF-β1. Therefore, we searched for differentially expressed components of the TGF-β signaling pathway in expanded chondrocytes, which may substantiate these observations. We focused on components involved in the immediate initiation and activation of the TGF-β signaling pathway, namely the TGF-β isoforms TGF-β1, TGF-β2, and TGF-β3 and the receptors TβR1 and TβR2.

**Fig 6:**
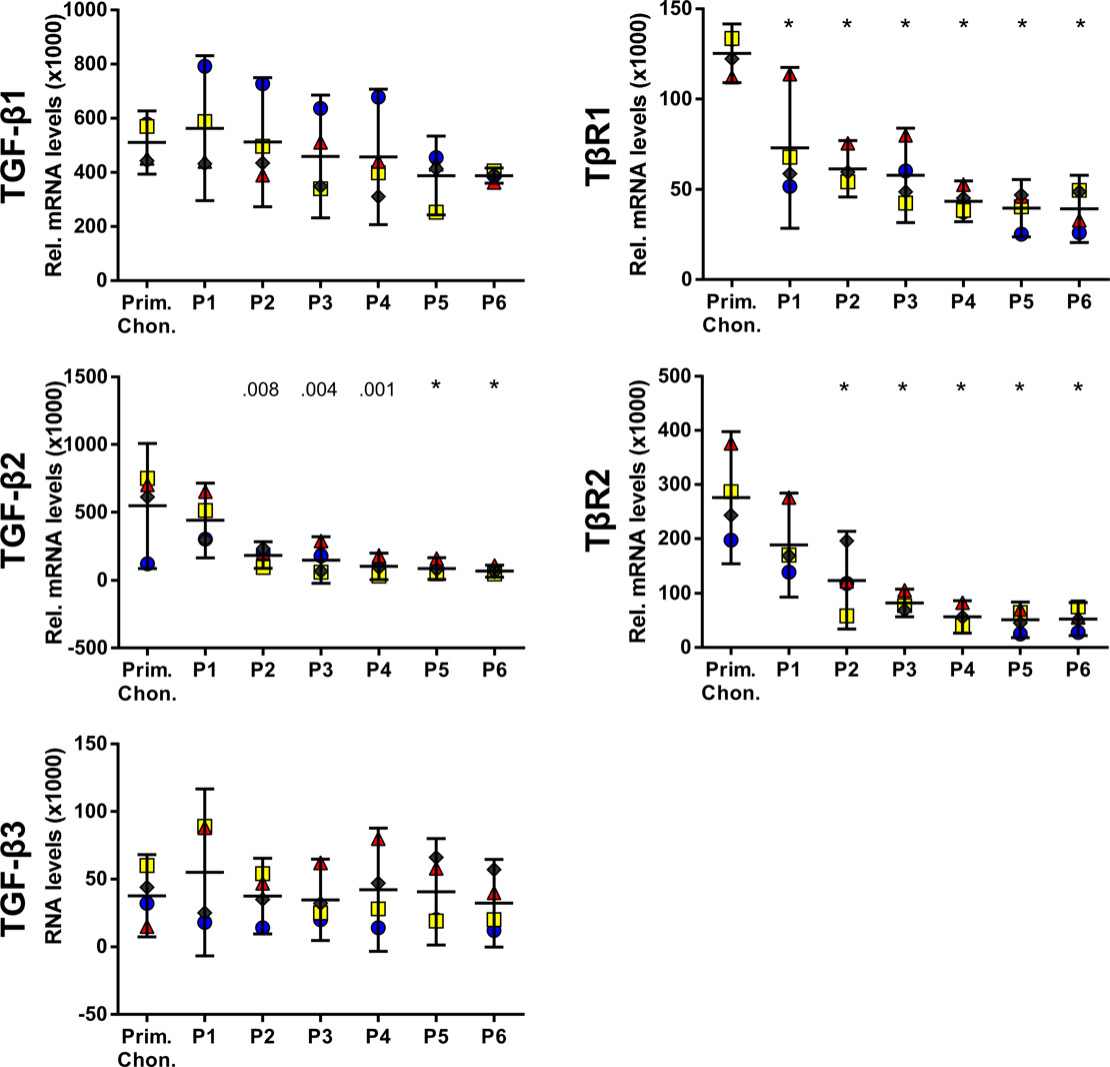
Levels of transcripts coding for TGF-β isoforms and TGF-β receptors in expanded chondrocytes. The levels of transcripts coding for TGF-β2 and TGF-β receptors decreased during expansion of articular chondrocytes, while those of TGF-β1 and TGF-β3 remained constant. Levels of transcripts were normalized to B2M and are presented as mean with the 95% confidence interval derived from four animals (illustrated with different symbols and colors). Levels of transcripts of P1–P6 were compared to the levels of primary chondrocytes (ANOVA), indicated by exact *P* values or * *P* < .001.

In primary chondrocytes, levels of transcripts coding for TGF-β1 and TGF-β2 were similar, whereas levels of TGF-β3 were approximately 10 times lower (Fig. 6). During expansion, the levels of transcripts coding for TGF-β1 and TGF-β3 showed slight variations, but remained within 30% of the levels in primary chondrocytes. In contrast, compared to primary chondrocytes, the levels of transcripts coding for TGF-β2, TβR1 and TβR2 were five-, three-, and five-fold decreased, respectively, at P4–P6.

## Discussion

For cell-based cartilage repair strategies such as ACI, *in vitro* expansion of primary chondrocytes is necessary to obtain the number of cells needed for therapy [47]. *In vitro*, chondrocytes expanded for only a short period of time maintain the potential to autonomously generate a chondroinstructive microenvironment and build cartilage-like tissue. The loss of chondrogenic potential may explain the failure of ACI to generate a stable long-lasting repair tissue *in vivo* within a cartilage defect [10, 11, 48]. In the present study, we showed that autonomous cartilage-like tissue formation relies on functional TGF-β signaling and propose that alterations in this pathway contribute to the loss of chondrogenic potential of expanded articular chondrocytes.

During the expansion of bovine articular chondrocytes in monolayer cultures, we found that the expression of COL2 decreased, while that of COL1 increased, an observation that has been published previously and has been described as dedifferentiation [9, 20, 49, 50]. These changes may represent the cells’ adaptation to the changes in the microenvironment from the native cartilage tissue to an *in vitro* culture system optimized to induce and maintain cell proliferation. We proceeded to assess the chondrogenic potential of bovine articular chondrocytes in high-density pellet cultures. Only chondrocytes grown for less than 5.2 (3.5–7.0, 95% CI) PD showed efficient autonomous formation of cartilage-like tissue. Similar observations have been reported in human articular chondrocytes collected from healthy knee joints (patient age 67.6 ± 1.9 years (mean ± SD)), in which a value of 3.57–4.19 PD was identified as the threshold for cells to maintain the potential for the autonomous formation of cartilage-like tissue [24]. Exogenous TGF-β1, known for its chondroinductive properties, has been shown by others [32, 51, 52] a nd in the present study to induce the formation of cartilage-like tissue in expanded chondrocytes that have lost the potential for autonomous cartilage formation. However, the tissue formed with the support of exogenous TGF-β1 is different from the tissue formed without TGF-β1, with cells expressing higher levels of COL1 and COL10. This mirrors the development of fibrocartilaginous tissue and of hypertrophic cartilage instead of the hyaline cartilage aimed for, which is in agreement with previous observations [51, 53]. Due to the importance of TGF-β signaling for cartilage formation [54, 55] we hypothesized that a possible alteration in this pathway in expanded chondrocytes renders them incompetent to form cartilage autonomously, but that signaling can be restored by the addition of exogenous TGF-β1.

As a first step to corroborate our hypothesis of the necessity for functional endogenous TGF-β signaling in the formation of cartilage-like tissue by expanded chondrocytes, the effect of the TβR1 kinase inhibitor SB-505124 on this process was analyzed. We showed that blocking TGF-β signaling abrogated in a dose-dependent manner the formation of a cartilage-like tissue in P1 chondrocytes, demonstrating this pathway as crucial for autonomous cartilage-like tissue formation. In a next step we searched for differentially expressed components of the TGF-β signaling pathway in expanded chondrocytes, which may mirror the observed dependence of autonomous cartilage-like tissue formation on this pathway. We focused on assessing these components in expanded chondrocytes, which most accurately reflects the phenotype of the cells at the initiation of the pellet cultures when the cells are required to generate a chondroinstructive microenvironment. A decline in the levels of TGF-β2 was found, while levels of TGF-β1 and TGF-β3 remained unchanged. There are two possible ways for these changes to contribute to the loss of autonomous cartilage formation: either by a reduction in the total amount of the TGF-β isoforms or by the loss of a specific activity induced by TGF-β2. Indeed, TGF-β2 is known as a chondrogenic growth factor for chondrocytes [56] and mesenchymal stromal cells [57] and it counteracts collagen degradation in cartilage explants [58].

We observed decreasing levels of transcripts encoding TβR1 and TβR2 during expansion in monolayer culture. In agreement with this observation, Bauge *et al*. [59] reported that in human articular chondrocytes isolated from femoral heads (median age of patients = 68 years) and cultured in monolayer cultures for three passages (corresponding to 4–5 PD) levels of mRNA encoding TβR2 was three-fold reduced as compared to primary chondrocytes. Transient overexpression of TβR2 for 24 hours in these passage three cells partially reversed the loss of the chondrogenic phenotype in cells in monolayer culture, as evidenced by increased levels of transcripts coding for COL2 and ACAN. Blaney Davidson *et al*. [38] reported lower expression levels of TGF-β receptors in aged mice that are prone to develop OA, further emphasizing the importance of TGF-β signaling for the maintenance of the chondrogenic phenotype and cartilage homeostasis. Furthermore, Serra *et al*. [60] showed that transgenic mice overexpressing a dominant negative form of TβR2 in skeletal tissue displayed progressive cartilage degradation and degenerative joint disease, resembling OA in humans, while Spagnoli *et al*. [61] reported an essential role of TβR2 in joint morphogenesis and skeletal development. Taking together, the reduced expression of TGF-β receptors, predominantly TβR2, may contribute to the lack of development of a cartilage-like tissue because of the decreased sensitivity and signal strength with respect to the autocrine and paracrine actions of cell-derived TGF-βs, which in addition are lower expressed (TGF-β2) in expanded chondrocytes. Addition of TGF-β1 (10 ng/ml) to the pellet cultures can compensate for the loss of the potential to form cartilage-like tissue autonomously. However, after prolonged expansion of the chondrocytes (P4 and thereafter), the efficiency of TGF-β1 to induce the formation of cartilage-like tissue is decreased as compared to P2 and P3, albeit an expression of transcripts encoding the TGF-β receptors similar to that in P3 was observed. Since the phospho-SMAD2/3 reporter assay showed similar responses in P3 and P6, we failed to find evidence for reduced sensitivity to TGF-β1 in cells from later passages. Our results indicate that in addition to exogenous TGF-β1, other factors are likely to be critically involved in the process of cartilage-like tissue formation by *in vitro* expanded cells after prolonged monolayer culture.

We showed that blocking TGF-β signaling using the TβR1 kinase inhibitor SB-505124 abrogated the formation of a cartilage-like tissue in P1 chondrocytes. Although the intensity of histological staining for glycosaminoglycans and COL2 was reduced when P1 pellet cultures were treated with increasing concentrations of the TβR1 inhibitor, the size of the pellet and cellular organization (lacunae, density), were not affected. These cultures, however, were morphologically distinct from P3 pellet cultures, which did not form cartilage-like tissue. These data led us to conclude that even though autocrine and paracrine TGF-β signaling is essential, its reduced activity, possibly through lower expression of TGF-β2 and TGF-β receptors during monolayer culture, is not the only factor determining the potential for autonomous cartilage-like tissue formation. Factors acting independently of the TGF-β signaling pathway during the induction of cartilage formation may still be active in articular chondrocytes from early passages (P1, P2) and may explain the different morphological appearance between the SB-505124 treated pellet cultures of P1 cells and the pellet cultures of P3 cells without exogenous TGF-β1. While the expression and activity of such factors may be lost during further expansion, it may be recovered and/or be compensated for upon supplementation of the cultures with TGF-β1. The identification of this/these factor(s), their interaction with the TGF-β signaling pathway, and detailed investigation of additional components of the TGF-β signaling pathway will further contribute to our understanding of the mechanisms by which expanded chondrocytes lose the potential to form cartilage-like tissue *in vitro*.

In conclusion, the data presented herein demonstrate a critical role for TGF-β signaling in autonomous cartilage formation *in vitro* by expanded bovine articular chondrocytes. Strategies to maintain the responsiveness of expanded chondrocytes to the autocrine and paracrine actions of chondrogenic growth factors merit further evaluation and may contribute to an improved outcome of autologous cell-based cartilage repair approaches.
